# Design of the Optimal Trial of Combination Therapy

**DOI:** 10.3389/fendo.2020.00168

**Published:** 2020-04-03

**Authors:** Anne R. Cappola

**Affiliations:** Division of Endocrinology, Diabetes, and Metabolism, Department of Medicine, Perelman School of Medicine at the University of Pennsylvania, Philadelphia, PA, United States

**Keywords:** levothyroxine, LT4, liothyronine, LT3, hypothyroidism, thyroid hormone replacement, clinical trial

## Abstract

Patients who take levothyroxine monotherapy to treat hypothyroidism frequently experience residual symptoms despite TSH testing at target levels. Trials have been conducted to evaluate the potential benefit of combination therapy with levothyroxine and liothyronine, though results have not consistently demonstrated benefit. In addition to randomization, placebo-control, and masking, four additional design choices to consider include the study population, dosing strategy for levothyroxine and liothyronine, primary and secondary outcome selection, and statistical power. A thoughtful design that considers these features will increase the likelihood that a combination trial will be considered definitive and finally resolve the important question of whether combination therapy with levothyroxine and liothyronine is a better thyroid replacement strategy than levothyroxine monotherapy.

## Introduction

Clinical trials are difficult to design and execute, requiring multiple choices that affect feasibility of trial conduct and generalizability of the results. The best clinical trials are valid, due to good design and execution, and novel, answering new questions that are clinically important. The ultimate goal of a clinical trial should be to change clinical practice.

Key features of clinical trial design include randomization, which minimizes confounding, and masking, which minimizes bias. Randomized controlled trials are the only study design to address causality, and they provide Level 1 evidence [1]. The shortcomings of clinical trials are that they are only able to address a specific question in a specific population, difficult to execute, and expensive.

The thyroid produces two major hormones, thyroxine (T4), and triiodothyronine (T3), in a 14:1 ratio. T4 has a longer half-life than T3 does, and the majority of circulating T3 is subsequently produced by peripheral deiodination of T4. A sodium salt of T4 that can be orally absorbed—levothyroxine (LT4)–was first introduced into the US market in 1949 for treatment of hypothyroidism. LT4 remains the recommended medication for thyroid hormone replacement today [2].

However, 35% of patients who are receiving recommended treatment with LT4 have impairment in psychological well-being [3]. In addition, patients who are taking LT4 therapy have higher free T4 and lower T3 levels than euthyroid counterparts who are not taking LT4 [4]. It has been suggested that LT4 therapy is not complete replacement therapy, and that therapy with a combination of LT4 and liothyronine (LT3) would better replicate normal thyroid physiology and therefore reduce hypothyroid symptoms. Although the clinical trials that have been performed to assess whether combination therapy with LT4 and LT3 is superior to LT4 alone have not demonstrated clear, consistent evidence to support combination therapy [2], this question remains unanswered in the eyes of many patients and clinicians, who point to design weaknesses in these trials.

The best clinical trials randomize treatment assignments, have a placebo or other control, and mask participants, investigators, and anyone assessing outcomes to the treatment assignment. Design of the “optimal” trial of combination therapy requires particular attention to four additional design features: the study population, dosing strategy for LT4 and LT3, primary and secondary outcome selection, and statistical power.

## Study Population

The selection of the study population of a clinical trial affects both the size of the trial and its generalizability. A larger impact is generally seen from interventions in patients with more severe disease, thereby reducing the number of patients needed to enroll in the trial. However, any selection criterion will impact the generalizability of results to patients who were excluded based on that criterion.

Four major decisions about inclusion and exclusion criteria affect the design of a trial of combination therapy. The first is whether patients should be restricted based on the LT4 dose at study entry. Patients who are taking a dose of LT4 that is below a full replacement dose (widely considered to be 1.6 mcg/kg/day) are likely to have residual thyroidal production of T4 and T3 and may not derive as much benefit from the addition of T3 as those who have no endogenous thyroid function. In addition, depending on whether a fixed dose or a fixed ratio of T3 is used, variability in the baseline LT4 dose may affect the feasibility of achieving similar LT4:LT3 ratios across the study population.

Second, whether enrollment should be limited to symptomatic patients or expanded to any LT4 user is an equally important design decision. This decision is largely impacted by whether the primary outcome is based on patient-reported or physiologic outcomes. Enrolling only patients who have residual symptoms despite taking LT4 doses considered adequate based on thyroid function testing increases the likelihood of detecting symptomatic benefit from combination therapy. Patients who feel good at baseline are unlikely to feel better with an alternative therapeutic regimen. However, if the primary outcome involves testing physiologic changes, this restriction would not be required.

A third consideration is the etiology of hypothyroidism: autoimmune or due to destruction or removal of the thyroid gland. Patients with an autoimmune etiology of hypothyroidism are more likely to have other autoimmune syndromes in addition, leading to a greater likelihood of symptoms attributable to non-thyroid conditions. Patients who had surgical removal of their thyroid due to structural disease do not have underlying autoimmune thyroid conditions and are therefore a more homogenous population. However, the group with hypothyroidism due to autoimmunity represents a larger section of the community of patients with hypothyroidism, impacting generalizability if this group were excluded.

The fourth decision is whether to incorporate low baseline T3 levels as an inclusion criterion. Obtaining an accurate measurement of serum T3 is challenging due to assay variability and biological variability in the setting of caloric restriction. An additional challenge to using T3 levels as an inclusion/exclusion criterion is the lack of knowledge about what level represents true T3 deficiency. The current T3 reference range lower limit represents the bottom 2.5% of the distribution of T3 levels in a population of individuals without thyroid disease. It is possible that patients above this range could still experience problems related to T3 deficiency. One caveat is that it seems unlikely that symptomatic patients with T3 levels in the upper half of the distribution of T3 levels are experiencing issues attributable to T3 deficiency.

## Dosing Strategy of LT4 and LT3: The Intervention

This will likely be the most critical design feature for the uptake of trial findings by endocrinologists. There are four possible dosing goals: fixed dosing of a physiologic ratio of LT4:LT3 without titration based on thyroid function testing, variable dosing to achieve a TSH level within a specific window (e.g., 0.5–2.5 mU/L), variable dosing to achieve free T4 and T3 levels within reference ranges (or a narrower window within these ranges) irrespective of the TSH level, or a combination approach titrating to both serum TSH and T3/T4 ratio. Each approach has its advantages and disadvantages. However, abandonment of TSH testing as a guiding parameter for dose strategy would be counter to years of precedent in which TSH has been accepted as the primary thyroid function test. Furthermore, if the combination therapy and control groups differ with respect to their TSH concentrations at study conclusion, critics will argue that any differences in outcome were due to either underdosing or overdosing in one of the groups, threatening study validity.

Since LT4 pills are color coded by dose, masked titration of LT4 will require either manufacture of multiple doses of LT4 without dye or overencapsulation of commercially available LT4 with difficult to break capsules. The 5 mcg dose of LT3 is the only available dose and an identical placebo can easily be manufactured. If additional dose formulations of LT3 or a sustained release LT3 preparation were to become available, a more physiologically refined combination LT4/LT3 intervention could be tested. Neither is available at this time.

If consistent levels of T3 are desired, either twice or three times daily dosing of LT3 is required [5], with twice daily dosing preferred for patient convenience. LT4 and LT3 differ in their absorption, with higher variability in LT4 than in LT3 absorption. This is a drawback to using a fixed ratio of LT4/LT3 without titration to any thyroid function tests.

## Primary and Secondary Outcomes

The primary outcome should be the efficacy outcome that will most influence clinical practice. At the completion of the trial, if the analysis shows no statistically significant difference between groups in the primary outcome, regardless of the secondary outcomes, the overall interpretation of the trial will be that combination therapy is not different from standard of care therapy.

Which efficacy outcomes will change clinical practice? The primary reason that patients pursue combination therapy is to ameliorate symptoms of hypothyroidism that can be found in a simple internet search [6]. Physicians also want their patients to feel better. For both of these groups, the change in thyroid symptom scores would be the best primary outcome. In addition, endocrinologists want to understand the physiologic effects of different regimens across the multiple physiologic systems affected by thyroid hormones, including metabolic, cardiovascular, cognitive and musculoskeletal outcomes. The best clinical trials tend to have “hard” clinical outcomes that represent discrete, clinically relevant events. There is no single primary outcome that will satisfy all of these conditions.

The primary outcome that will affect the broadest group of individuals is one that assesses thyroid-related symptoms. Multiple questionnaires exist, though in a systematic review of quality of thyroid-specific health-related quality-of-life instruments, the Thyroid-Related Quality of Life Patient-Reported Outcome (ThyPRO) questionnaire was recommended for patients with hypothyroidism [7]. Validated versions include the full 85-item ThyPRO [8–12] and the 39-item ThyPRO-39 scale with the 22-item ThyPRO Composite QOL scale [13]. The ThyPRO-39 Composite QOL scale is based on 22 items from the Tiredness, Cognition, Anxiety, Depressivity, Emotional Susceptibility, Impaired Social Life, Impaired Daily Life and Overall QOL scales of ThyPRO. Item responses are scored 0 for “Not at all,” 1 for “A little,” 2 for “Some,” 3 for “Quite a bit,” and 4 for “Very much”/”Completely.”

Questionnaires may not adequately capture the elements that underlie patient preference. All studies should include assessment of patient preference for the randomized regimen over the LT4 regimen prior to randomization, as well as whether the patient believed they were randomized to combination or standard LT4 therapy. Maintenance of masking is critical for ensuring that the assessment of patient preference is not biased.

Secondary, physiologic outcomes should be selected based on the following properties: responsivity to changes in T3, participant burden, availability at multiple centers, standardization across centers, frequency of measurement, and cost. Across each domain of physiologic outcomes, there is a range of testing available for use in the clinical research setting. Possible tests of metabolic efficacy include objective measurements of weight and waist circumference, resting energy expenditure measured with a metabolic cart, and use of actigraphy for activity monitoring. For cardiovascular efficacy, tests include a lipid profile, resting pulse rate, blood pressure, echocardiogram, brachial artery flow mediated vasodilation, VO2 max testing, and measurement of carotid intimal medial thickness. Assessments of cognition should include tests of executive function and memory that are easy to administer. An example is the fluid cognition composite score of the NIH Toolbox cognition battery [14]. Potential musculoskeletal efficacy outcomes include bone biomarkers (e.g., C-telopeptide), DXA scans for bone density and body composition, hand grip strength, and tests of physical function, such as the Short Physical Performance Battery (SPPB) or the 400 meter walk test.

Safety assessments are also necessary in any trial of combination therapy. The same issues of anticipated relationship to LT3 use, participant burden, and cost to measure are as relevant for safety outcomes as for efficacy outcomes. Changes in symptoms of thyrotoxicosis should be specifically assessed in addition to a general assessment of adverse events. The primary concerns with LT3 are due to the chronotropic effects of T3 and include sinus tachycardia and atrial arrhythmias. The incidence of atrial fibrillation should be collected through self-report and EKG assessments at visits. Wearable 2-week cardiac monitors are now available at a reasonable cost that have the advantages of smaller size and better portability than Holter monitors.

## Statistical Power

The primary outcome of a study has to be frequent enough and the variation small enough to demonstrate a statistically significant and clinically significant difference. Enriching the study population with a higher risk group, for example, those taking higher replacement doses of LT4, allows for a smaller sample size. The primary and secondary outcomes must be prespecified in the trial protocol and a clinical trials registry prior to study enrollment. The trial protocol should also include prespecified subgroup analyses, such as by etiology of hypothyroidism, baseline T3 level, or genetic background (type 2 deiodinase or MCT8 single nucleotide polymorphisms). It should also include techniques for management of statistical significance in the face of comparisons across multiple outcomes, possibly including hierarchical analysis of secondary outcomes.

One possible outcome of a combination therapy trial is that there is no statistically significant difference between combination therapy and standard LT4 therapy. This will raise questions about acceptance of the results of a null trial. Any combination therapy trial should be adequately powered to detect the minimal clinically important difference (MCID), not just a statistically significant difference. The MCID can be difficult to determine and usually requires confirmation via validation studies. It is important that the primary outcome has a widely accepted MCID to ensure that null results are accepted as readily as positive findings.

## Discussion

### Recommendations for Design of the “Optimal” Trial

Patients taking LT4 doses close to the anticipated full replacement dose, at least above 1.2 mcg/kg/day, that is at least 100 mcg/day should be enrolled, both for feasibility of combination dosing at a physiologic T4:T3 ratio and for increased likelihood of benefit in those with less endogenous thyroid function. They should also have symptoms that are potentially attributable to hypothyroidism, since symptomatic improvement will be the primary driver of the clinical impact of combination therapy. Identification of individuals, based on symptoms or findings, who may actually benefit from LT3 is critical. Enrollment of patients with low or no symptom burden is an area of criticism of previous trials of combination therapy. TSH concentrations should be within the reference range at baseline, demonstrating appropriate control on LT4 therapy prior to enrollment. Including both patients with autoimmune thyroid disease and those with hypothyroidism due to removal or destruction of the thyroid would be ideal, with stratification of the analysis by group. Study budget would be a key consideration for this strategy. Baseline T3 levels are not likely to be helpful for determining study inclusion, though they should be evaluated in a subgroup analysis.

Physiologic dosing of T3 should be used in twice daily doses, for example, replacing 25 mcg of LT4 with 5 mcg of LT3 twice daily. Although this will result in a different ratio of LT4 to LT3 dosing in an individual whose baseline LT4 dose is 100 mcg vs. 200 mcg, with the currently available LT3 doses and the logistics of placebo control, this is the most feasible option for starting dose. Maintenance of masking, either through generation of identical placebos or overencapsulation, is important for maintaining the study integrity. At the end of the study, while still masked, participants should be asked to guess the therapy to which they were assigned and their preference compared with their LT4 regimen prior to study initiation.

The primary outcome should be a symptoms assessment, such as one of the ThyPRO questionnaires, and a limited battery of secondary efficacy outcomes should be performed, such as weight, lipid panel, resting heart rate, a cognitive battery, and bone biomarkers. These would require limited equipment and training to execute. Additional measures to consider are resting energy expenditure and a DXA scan for bone and body composition. Assessments of safety should include hyperthyroid symptoms, tachyarrhythmias, and adverse events. All assessments should be performed at baseline, shortly after randomization, and repeated over the course of the trial to assess early and sustained responses. The duration of the trial should be at least 1 year in order to assess persistence of efficacy and safety over sufficient duration. A parallel design is preferred due the long study duration and concerns about carryover effects and the impact of dropouts in a crossover design. Subgroup analyses, such as by genetic polymorphisms, should be prespecified.

### Additional Trial Considerations

The optimal clinical trial of combination therapy will require substantial funds and a significant commitment from study investigators and participants to implement. There will likely only be one large trial funded to answer this question. Design choices affect the validity and generalizability of a trial, which in turn affect the interpretation and clinical impact of the results. A thoughtful design that considers the features outlined above will increase the likelihood that a combination trial will be considered definitive and finally resolve the important question of whether combination therapy with LT4 and LT3 is a better thyroid replacement strategy than our current standard of care of LT4 monotherapy. If the clinical trial fails to show superiority of one of the regimens, then additional considerations, such as ease of adherence and cost should be incorporated into therapeutic recommendations.

## Author Contributions

AC drafted and edited the manuscript.

### Conflict of Interest

The author declares that the research was conducted in the absence of any commercial or financial relationships that could be construed as a potential conflict of interest.
